# Circular RNAs in Cardiovascular Diseases: Molecular Mechanisms, Therapeutic Advances, and Innovations

**DOI:** 10.3390/genes15111423

**Published:** 2024-10-31

**Authors:** Zheng Yuan, Shaoyuan Huang, Xin Jin, Shanshan Li

**Affiliations:** 1 College of Science, China University of Petroleum-Beijing, Beijing 102249, China; 2 School of Medicine, Nankai University, Tianjin 300071, China; 2120231765@mail.nankai.edu.cn (S.H.); xin.jin@nankai.edu.cn (X.J.)

**Keywords:** circRNA, cardiovascular diseases, synthetic circRNA sponges, RNA delivery systems

## Abstract

Circular RNAs (circRNAs) have emerged as promising therapeutic targets due to their unique covalently closed-loop structures and their regulatory roles in gene expression. Despite their potential, challenges in circRNA-based therapies include ensuring stability, tissue specificity, and efficient intracellular delivery. This review explores the implications of circRNAs in cardiovascular diseases (CVDs), providing an overview of their biogenesis, molecular mechanisms, and roles in disease pathology. In addition to discussing molecular features, this review highlights therapeutic advances, including small-molecule drugs targeting circRNAs, synthetic circRNA sponges, and innovations in drug delivery systems that enhance the effectiveness of these therapies. Finally, current challenges and future directions are addressed, emphasizing the need for continued research to fully unlock the therapeutic potential of circRNA-based strategies in cardiovascular medicine.

## 1. Introduction

Cardiovascular diseases (CVDs) continue to be the leading cause of mortality worldwide, accounting for approximately one-third of global deaths, despite significant advances in prevention, diagnosis, and treatment over the past several decades [1]. Traditional risk factors, including hypertension, smoking, dyslipidemia, obesity, diabetes, poor diet, and excessive alcohol consumption, are well-established contributors to the onset and progression of CVDs [2]. The rising prevalence of CVDs in younger populations signals an urgent need for novel biomarkers and therapeutic strategies to improve diagnostic precision and treatment outcomes [3]. This epidemiological shift underscores the importance of developing innovative approaches to reduce CVD incidence and associated mortality.

In recent years, non-coding RNAs have gained considerable attention in cardiovascular research [4]. CircRNAs, first discovered in 1971, form covalently closed-loop structures through back-splicing, which makes them highly stable and resistant to degradation [5]. Their biological roles have come into focus with the advancement of RNA sequencing technologies, revealing their involvement in key regulatory processes such as miRNA sponging, RNA-binding protein interactions, and gene expression modulation [6]. Given their stability and regulatory versatility, circRNAs have emerged as potential biomarkers and therapeutic targets in CVDs [7]. Aberrant circRNA expression has been implicated in key pathological processes underlying CVDs, including cardiac hypertrophy, atherosclerosis, myocardial infarction, heart failure, and pulmonary hypertension [8,9,10,11,12].

This review provides a focused examination of circRNAs in CVDs, including their biogenesis, molecular mechanisms, and functional roles. It also evaluates their potential as diagnostic and prognostic biomarkers and explores therapeutic advancements, such as circRNA sponges and small-molecule drugs. Finally, innovations in delivery systems that enhance the specificity and efficacy of circRNA-based therapies are discussed.

## 2. Biogenesis and Functions of CircRNA

### 2.1. Models of CircRNA Biogenesis

**Figure 1:**
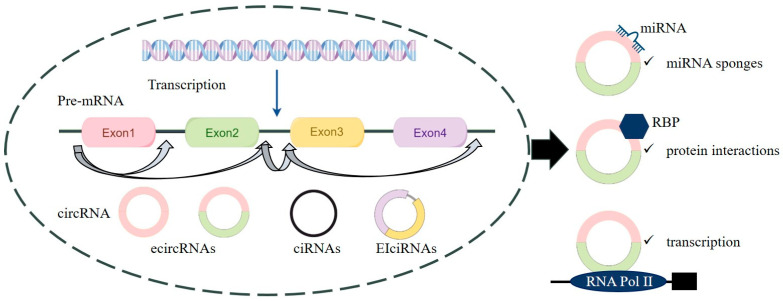
Biogenesis and functions of circRNA.

CircRNAs are classified into three types: exon-derived circRNAs (ecircRNAs), intron-derived ciRNAs, and exon–intron circular RNAs (EIciRNAs) (Figure 1). EcircRNAs, the most common, are increasingly detected in human cells through RNA sequencing techniques [13,14]. CircRNA biogenesis is explained by several models. In exon skipping, non-adjacent exons form a lariat structure that is spliced into a stable circRNA. In intron-pairing-driven circularization, complementary intronic sequences bring splice sites together, enabling back-splicing [15]. This process is regulated by conserved motifs, like GU-rich and C-rich elements, which also protect circRNAs from degradation. CircRNAs are formed when a lariat intron escapes debranching during splicing, and its 3′ tail is cleaved to create a stable structure [16]. RNA-binding proteins (RBPs) further facilitate circRNA formation by bringing splice sites into proximity through protein interactions. CircRNAs’ lack of poly-A tails and 5′ caps makes them highly resistant to exonuclease degradation, leading to their exceptional stability, especially in the cytoplasm [17]. This inherent stability, along with resistance to degradation, positions circRNAs as promising candidates for biomarkers in disease diagnostics and as therapeutic targets, particularly in cardiovascular diseases.

### 2.2. Functions of CircRNAs

One of the most well-established functions of circRNAs is their ability to act as miRNA sponges, where they bind and sequester miRNAs, thereby preventing their interaction with target mRNAs. This regulatory mechanism modulates gene expression by controlling the availability of miRNAs [18]. The capacity of circRNAs to function as miRNA sponges has been implicated in a variety of diseases, including cardiovascular disorders, and presents significant potential for therapeutic applications. CircRNAs play a crucial role in protein interactions by binding specific proteins to regulate their activity or sequester them from their functional sites [19]. Additionally, circRNAs can act as protein reservoirs, releasing bound proteins in response to specific conditions [20]. Emerging evidence suggests that some circRNAs may directly participate in protein translation [21]. CircRNAs with internal ribosome entry sites (IRESs) or m6A modifications can recruit ribosomes to translate proteins [22], which has opened new avenues in understanding how circRNAs may contribute to cellular processes beyond their traditional non-coding roles. Recent studies demonstrate that exogenous circRNAs can drive robust and stable protein expression in eukaryotic cells, highlighting their potential as alternatives to linear mRNA in therapeutic applications [23]. The discovery of circRNA-driven protein translation offers new insights into the therapeutic manipulation of circRNAs in diseases such as cardiovascular disorders.

## 3. The Role of CircRNAs in CVDs

### 3.1. CircRNAs in Cardiomyocyte Apoptosis and Repair

Cardiomyocyte apoptosis is a critical process in the development and progression of various CVDs, including myocardial infarction, heart failure, and ischemia-reperfusion (I/R) injury. As research on circRNAs has expanded, their role in regulating cardiomyocyte apoptosis and promoting repair has become increasingly evident. One of the primary mechanisms by which circRNAs influence cardiomyocyte apoptosis is through their function as miRNA sponges, modulating the expression of apoptosis-related genes. For example, circSAMD4A has been shown to sequester miR-138-5p, leading to increased apoptosis and inflammation during hypoxia/reoxygenation (H/R) injury [24]. This finding suggests that circSAMD4A may have therapeutic potential in mitigating cardiac injury caused by H/R, though further research is needed to confirm its efficacy and relevance in clinical settings. Similarly, circ-ITCH exerts a protective effect against myocardial apoptosis by acting as a sponge for miR-17-5p, thereby inhibiting the Wnt/β-catenin signaling pathway [25]. These studies highlight the pivotal role of circRNAs in modulating apoptosis and suggest their potential as therapeutic targets for reducing cardiomyocyte death and improving outcomes in CVDs. Another prominent example of a circRNA involved in cardiomyocyte apoptosis during ischemic injury is circNCX1. CircNCX1 plays a critical role in oxidative-stress-induced cardiomyocyte apoptosis during ischemic myocardial injury by acting as a sponge for miR-133a-3p, leading to the upregulation of the pro-apoptotic gene CDIP1. The knockdown of circNCX1 has been shown to reduce CDIP1 levels and attenuate apoptosis [26]. This highlights the potential of circNCX1 as a key regulator in apoptosis pathways and its value in developing therapies to limit ischemic injury in cardiovascular diseases. Mitochondrial dysfunction is a key factor in cardiomyocyte apoptosis, and circRNAs have been shown to regulate mitochondrial dynamics. Wang et al. [27] identified MFACR, a circRNA that stimulates mitochondrial division and apoptosis. Knocking down MFACR reduced myocardial infarction size and improved cardiac function, highlighting its potential as a therapeutic target for preventing mitochondrial-induced cardiomyocyte apoptosis.

In addition to its role in apoptosis, Circ-Amotl1 has been shown to enhance cardiomyocyte survival and promote myocardial regeneration by activating the Akt signaling pathway, improving cell survival and repair following doxorubicin (DOX)-induced cardiomyopathy [28]. Similarly, circPan3, downregulated by miR-31-5p during DOX treatment, exacerbates cardiotoxicity through the miR-31-5p/QKI axis. Targeting circPan3 could mitigate DOX-induced cardiomyopathy [29]. These findings underscore the potential of circRNAs as key players in cardiac regeneration and repair, offering new therapeutic avenues for cardiovascular diseases. While the role of circRNAs in cardiomyocyte apoptosis and repair is promising, current studies face limitations, including reliance on animal models and in vitro experiments that may not fully reflect human cardiovascular disease.

### 3.2. Cardiac Hypertrophy

Cardiac hypertrophy is an adaptive response that enables the heart to maintain adequate cardiac output under increased workload or injury [30]. However, sustained hypertrophic stimuli, such as chronic pressure overload, can result in pathological hypertrophy, ultimately progressing to heart failure [31,32]. Recent studies have found the involvement of circRNAs in the regulation of cardiac hypertrophy, particularly through their function as miRNA sponges that modulate key molecular pathways. CircSlc8a1, derived from exon 2 of the Slc8a1 gene, has been studied in cardiac hypertrophy [33]. Lim et al. [34] used a biotin-labeled probe in RNA pull-down experiments and luciferase assays to show that circSlc8a1 sponges miR-133, a key regulator of cardiac remodeling. In vivo, AAV9-mediated circSlc8a1 knockdown in a transverse aortic constriction (TAC) model reduced cardiac hypertrophy by upregulating hypertrophy-related genes [35]. CircRNA_000203 increases cardiac hypertrophy by modulating miRNA interactions that regulate hypertrophic gene expression. Li et al. [18] found that circRNA_000203 is upregulated in Angiotensin II-induced hypertrophic mouse myocardium and neonatal mouse ventricular myocytes, leading to increased cell size and hypertrophic markers. Wang et al. [36] found the involvement of circHRCR as a protective factor that mitigates hypertrophy by sponging miR-223, a key promoter of hypertrophic signaling.

### 3.3. Myocardial Infarction

Myocardial infarction (MI) remains one of the leading causes of mortality worldwide, posing a significant global health burden as one of the most prevalent and deadly diseases [37,38]. MI occurs when coronary arteries are blocked by atherosclerotic plaque, leading to ischemia, hypoxia, and the necrosis of myocardial tissue [39]. In recent years, circRNAs have emerged as key regulators in the pathophysiology of MI [40,41]. CircFNDC3b promotes cardiac repair following MI [42]. Garikipati et al. [42] found that circFNDC3b was significantly downregulated in both a mouse MI model and patients with myocardial ischemia. The overexpression of circFNDC3b via AAV9-mediated gene delivery improved left ventricular function and reduced ischemic damage in mice. Mechanistically, circFNDC3b sponges miRNAs such as mmu-miR-93-3p, mmu-miR-412-3p, and mmu-miR-298-5p, which regulate heme oxygenase-1 (HO-1) and vascular endothelial growth factor (VEGF), promoting neovascularization and cardiac repair. CircNfix has also been shown to regulate cardiac regeneration after MI. Research demonstrated that circNfix is controlled by a super-enhancer, a cis-acting element that regulates genes involved in cardiac regeneration. The downregulation of circNfix promotes cardiomyocyte proliferation and angiogenesis while inhibiting apoptosis [43], suggesting that targeting circNfix could be a promising strategy to enhance cardiac regeneration post MI.

### 3.4. Myocardial Fibrosis

Myocardial fibrosis, a hallmark of many heart diseases, is characterized by the excessive accumulation of extracellular matrix (ECM) proteins in the myocardium, leading to increased stiffness, impaired cardiac function, and progression to heart failure [44,45]. This fibrotic process is driven by various factors, including inflammatory cytokines, reactive oxygen species, mast-cell-derived proteases, endothelin-1, and the renin–angiotensin–aldosterone system. Key growth factors such as transforming growth factor-β (TGF-β) and platelet-derived growth factor (PDGF) also play crucial roles in fibroblast activation and ECM deposition [46,47]. Recent studies have highlighted the role of circRNAs in regulating myocardial fibrosis, offering novel therapeutic targets for this condition [48]. CircRNA_000203 plays a critical role in myocardial fibrosis by upregulating key fibrosis markers. Tang et al. [49] showed that circRNA_000203 acts as a miR-26b-5p sponge, inhibiting miR-26b-5p’s regulation of Col1a2 and CTGF, which are essential for ECM production. In addition, circNFIB can attenuate myocardial fibrosis [50]. Zhu et al. [19] found that circNFIB expression was reduced in post-MI mouse models and cardiac fibroblasts treated with TGF-β, a driver of fibrosis. Acting as an miR-433 sponge, circNFIB regulates pro-fibrotic effects. The overexpression of circNFIB reduced fibroblast proliferation and differentiation, reversing miR-433’s fibrotic effects [19]. The circNFIB–miR-433 axis offers a potential therapeutic target for myocardial fibrosis.

### 3.5. CircRNAs in Heart Failure

Heart failure (HF) is a common complication following acute myocardial infarction (MI), with many patients progressing to chronic HF due to worsening left ventricular (LV) dysfunction [51,52,53]. The identification of biomarkers that can predict HF onset post MI is essential for personalized healthcare, enabling earlier interventions and tailored treatment strategies [54,55]. MICRA (myocardial-infarction-associated circular RNA) has emerged as a promising biomarker for predicting heart failure (HF) development post myocardial infarction (MI) [56]. A study [57] identified a significant inverse relationship between MICRA levels and ejection fraction (EF), suggesting its value as an early indicator of left ventricular dysfunction. Despite its potential, the exact mechanisms by which MICRA contributes to HF progression and LV remodeling remain unclear.

**Figure 2:**
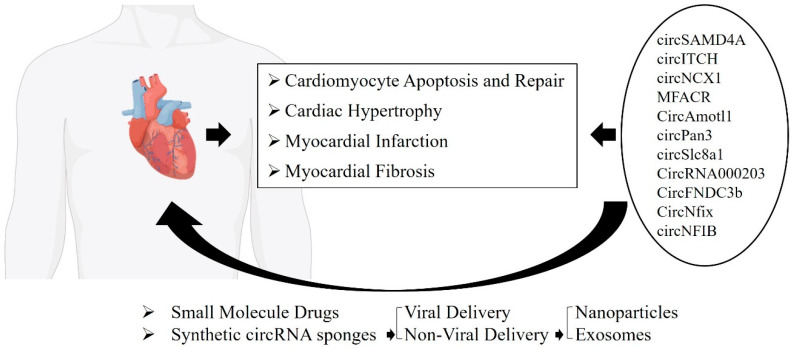
Circular RNAs in cardiomyopathies, therapeutic advances, and drug delivery innovations (Table 1).

These studies underscore the significant role of circRNAs in regulating various cardiac pathophysiological processes, positioning them as promising therapeutic targets for treating cardiac disorders (Figure 2). However, the majority of the research has been conducted in animal models, raising concerns about the applicability of these findings in human clinical settings. To fully harness the therapeutic potential of circRNAs, future research must focus on bridging the gap between preclinical studies and clinical applications. In conclusion, circRNAs offer great promise as therapeutic targets for cardiac diseases, but realizing their potential will require a deeper understanding of their molecular functions coupled with advancements in delivery technologies to ensure efficient and targeted therapeutic outcomes in cardiovascular medicine.

## 4. Design of Potential Small-Molecule Drugs Targeting CircRNAs

Small-molecule drugs, characterized by their low molecular weight (less than 900 daltons), have long been a cornerstone of pharmacotherapy due to their ability to diffuse across cell membranes, interact with intracellular targets, and provide oral bioavailability [58]. In the emerging field of RNA-based therapeutics, these drugs are particularly promising for modulating specific RNA structures [59]. Small molecules can be designed to selectively bind to distinct RNA motifs, such as hairpins, loops, and bulges found in circRNAs. By targeting these RNA structures, small molecules can disrupt critical RNA–protein or RNA–RNA interactions [60]. This capability is significant for treating diseases associated with dysregulated circRNAs. Unlike traditional therapies that target downstream protein effects, this precision medicine approach aims to correct aberrant circRNA functions directly, thereby addressing the root causes of disease with enhanced specificity.

### 4.1. Advantages of Small-Molecule Drugs in Targeting RNA Molecules

Small-molecule drugs present several key advantages for targeting RNA molecules, particularly circRNAs. (1) Selective Targeting: Small molecules can be engineered to bind to specific RNA structures such as hairpins, loops, or bulges in circRNAs. This allows for the precise modulation of disease-related circRNAs while minimizing off-target effects on other RNA molecules [61]. (2) Intracellular Accessibility: Their small size and lipophilic nature enable small molecules to easily penetrate cell membranes, reaching intracellular targets, including cytoplasmic circRNAs, where many disease-related pathways are regulated. (3) Oral Bioavailability: Unlike larger biologics or RNA-based therapies such as antisense oligonucleotides, many small molecules are orally bioavailable, offering a more convenient and practical option for chronic disease management [62]. (4) Regulation of RNA–Protein Interactions: CircRNAs often function by interacting with miRNAs or RNA-binding proteins (RBPs). Small molecules can be designed to disrupt or enhance these interactions, providing a powerful tool for modulating circRNA-related regulatory networks involved in disease processes.

### 4.2. Potential Mechanisms of Targeting CircRNAs with Small Molecules

Small-molecule drugs can target circRNAs through various mechanisms, leveraging their structural and functional features. Here are several possible mechanisms. (1) The Disruption of miRNA Sponging: CircRNAs often act as miRNA sponges, sequestering miRNAs and preventing them from binding to their target mRNAs. Small molecules can bind to miRNA-binding sites on circRNAs, disrupting this interaction and allowing miRNAs to regulate their target genes. (2) The Modulation of RNA–Protein Interactions: CircRNAs frequently interact with RNA-binding proteins (RBPs), affecting splicing, translation, and gene expression. Small molecules can alter these interactions by binding to either the circRNA or the RBP, impacting the function of the circRNA–RBP complex. (3) The Direct Modulation of RNA Structure: Small molecules can bind directly to circRNA structures, inducing conformational changes that affect their function. By stabilizing or destabilizing specific RNA motifs, these molecules can modulate the regulatory roles of circRNAs in various biological processes.

High binding affinity is essential for small molecules to effectively target RNAs. This is achieved through structure-based design, where small molecules are engineered to fit into specific pockets or grooves in the RNA structure. Key interactions include hydrogen bonding, pi-stacking, and van der Waals forces, which can be optimized to enhance binding affinity. Ensuring specificity is crucial to avoid off-target effects. RNA molecules often share structural similarities, making it challenging to design small molecules that selectively bind to specific RNA sequences. Structure–activity relationship (SAR) studies and high-throughput screening are vital for identifying small molecules that specifically target circRNA motifs without affecting other RNA species. The unique circular structure of circRNAs further aids in improving specificity compared to linear RNAs.

### 4.3. Advances in Small-Molecule Drugs Targeting RNA

The development of small-molecule drugs targeting RNA is still in its early stages, particularly in the context of CVDs. However, several pioneering drugs have demonstrated the potential of this approach in other conditions. One notable example is risdiplam, which is approved for treating spinal muscular atrophy (SMA). Risdiplam functions by modulating the pre-mRNA splicing of the SMN2 gene, thereby enhancing the production of the functional SMN protein [63]. This strategy addresses the underlying genetic defect in SMA, showcasing significant therapeutic success [64]. Similarly, branaplam, an investigational drug for SMA, also targets RNA splicing. Branaplam promotes the inclusion of exon 7 in SMN2 pre-mRNA, leading to increased levels of the functional SMN protein [65]. This RNA-targeting strategy underscores the therapeutic potential of modulating RNA splicing to address genetic disorders at their molecular roots [66]. RG7916, another RNA-targeting drug for SMA, binds to specific RNA sequences and modulates splicing to enhance therapeutic protein production [67]. These examples illustrate the potential of small molecules to regulate RNA processing for therapeutic applications. Although these examples come from the treatment of SMA and other genetic disorders, they provide a valuable proof of concept for the future use of RNA-targeting small molecules in other diseases, including cardiovascular conditions. However, it is important to note that, as of now, no small molecule targeting RNA has been specifically developed to correct single gene defects in CVDs. The success of RNA-targeting therapies in SMA suggests that similar approaches could eventually be applied to cardiovascular diseases as research advances. In response to recent findings and the current landscape, we emphasize that the therapeutic potential of RNA-targeting small molecules in cardiovascular diseases is still hypothetical and requires further validation through both preclinical and clinical studies. The examples provided serve to illustrate the future promise of this therapeutic strategy rather than its current application in cardiovascular diseases.

## 5. Synthetic CircRNA Sponges for Future Therapeutic Application

Natural circRNAs possess two key properties that make them promising candidates for therapeutic applications: their resistance to exonuclease degradation and their ability to regulate miRNA function by binding specific miRNAs [68]. Leveraging these properties, researchers have begun synthesizing circRNA sponges that can selectively bind and inhibit specific miRNAs, offering a novel approach to disease treatment.

One early example is scRNA21, a synthetic circRNA designed to bind miR-21 in vitro [69]. MiR-21, an oncogenic miRNA implicated in various cancers, including gastric cancer, was effectively sequestered by scRNA21. By sponging miR-21, scRNA21 significantly reduced gastric cancer cell proliferation, demonstrating the potential of synthetic circRNA sponges for miRNA inhibition in cancer therapy. Another study developed a circular RNA with binding sites for both miR-21 and miR-93, creating a multi-miRNA sponge [70]. This synthetic circRNA effectively inhibited endogenous miR-21 and miR-93, which are implicated in esophageal carcinoma, offering a multi-target approach to inhibit multiple oncogenic miRNAs simultaneously.

Although much of the current research on synthetic circRNA sponges has focused on cancer, promising developments are now emerging in the context of CVDs. A recent study introduced a novel therapeutic approach for CVDs, in which a synthetic circmiR sponge was designed to target pro-hypertrophic miRNAs. This synthetic circmiR was delivered to cardiomyocytes in vivo using adeno-associated viruses (AAVs) in a transverse aortic constriction (TAC) mouse model of cardiac hypertrophy. The treatment successfully attenuated hypertrophic disease characteristics and preserved cardiac function [71].

These findings highlight the potential of synthetic circRNA sponges as novel therapeutic tools for cardiovascular diseases. While the field is still in its early stages, the successful use of circmiRs to mitigate cardiac injury and preserve function in animal models offers an exciting glimpse into the future of circRNA-based therapeutics. As more research focuses on optimizing these sponges for cardiovascular applications, they may provide a promising therapeutic strategy for conditions like myocardial fibrosis, heart failure, and other cardiovascular pathologies where miRNAs play a critical role.

### 5.1. Synthetic Strategy

The synthesis of circRNAs in vitro involves a variety of methods, including enzymatic ligation, chemical ligation, and ribozymes. These techniques aim to efficiently promote RNA cyclization, allowing for the creation of functional circRNAs with therapeutic potential [71].

Enzymatic ligation is a widely utilized method for generating circRNAs in vitro [72]. This process typically begins with the transcription of RNA using T7 RNA polymerase, which employs linearized DNA as a template. Following RNA synthesis, a deoxyoligonucleotide bridge is employed to join the 3′-phosphate (p) and 5′-hydroxyl (OH) ends of the RNA molecule, facilitating the circularization process. T4 ligases, including T4 DNA ligase, T4 RNA ligase I, and T4 RNA ligase II, are commonly used enzymes for this purpose [72,73]. These ligases catalyze the joining of the 5′ and 3′ ends of the RNA, either directly or through a DNA or RNA bridge. After the ligation reaction, the circular RNA product is typically purified and isolated. This method has been effectively employed to synthesize small RNA molecules, generally less than 500 nucleotides in length. For instance, single-stranded circular miRNA sponges have been successfully synthesized using enzyme-mediated RNA cyclization [74]. However, the efficiency of T4 RNA ligase I tends to decline for larger RNA molecules, making it less suitable for synthesizing macromolecules [75]. This reduced efficiency is likely attributable to steric hindrance and diminished enzyme activity when dealing with larger, more complex RNA structures.

To overcome the limitations of enzymatic ligation, particularly for larger circRNAs, chemical ligation strategies have been explored. Chemical methods involve the formation of covalent bonds between RNA ends using chemical reagents that can promote cyclization without relying on enzymatic activity [72]. Chemical ligation offers advantages in the synthesis of larger RNA macromolecules, as it is less prone to the inefficiencies encountered with enzymatic ligation when dealing with longer RNA sequences. Although chemical strategies provide an alternative to enzyme-based ligation, further studies are needed to determine whether they can match or surpass the efficiency of enzymatic methods, especially for therapeutic applications. Investigating the competitiveness and efficiency of chemical ligation compared to enzymatic approaches could pave the way for more efficient RNA synthesis methods for larger molecules [76]. In summary, enzymatic ligation is a robust method for generating circRNAs, especially for small to medium-sized RNA molecules. However, the growing need for larger circRNAs may drive the adoption of chemical ligation strategies to address the limitations of enzymatic methods. Future research comparing the efficiency and applicability of these approaches will be essential for optimizing the synthesis of therapeutic circRNAs.

### 5.2. Functional Efficiency and Challenges of Synthetic CircRNA Sponges

The synthesis of circRNAs in vitro involves the careful consideration of factors such as RNA size and production site (in vitro or in vivo), which affect the efficiency and functionality of synthetic circRNA sponges [77]. These sponges work by competing with miRNAs, thereby preventing miRNAs from binding to their target mRNAs and inhibiting post-translational gene regulation. This competition enables circRNA sponges to modulate target gene expression by reducing miRNA-mediated repression. Additionally, circRNAs’ circular structure confers higher stability compared to linear RNAs, making them more resistant to exonuclease degradation and thus more suitable for therapeutic applications requiring prolonged activity.

CircRNAs bind to miRNAs with approximately ten times greater efficiency than other transcripts. This heightened binding efficacy stems from the multi-site binding potential of circRNAs, enabling them to function effectively as miRNA sponges. By incorporating multiple miRNA binding sites, synthetic circRNA sponges can sequester miRNAs more effectively, reducing their interaction with target mRNAs, and thus modulating the expression of genes associated with disease. This functional efficiency makes circRNA sponges effective tools for regulating miRNA activity, especially in diseases with critical miRNA dysregulation. In cardiovascular diseases, where miRNAs regulate processes like cardiac hypertrophy, myocardial fibrosis, and heart failure, synthetic circRNA sponges could mitigate disease progression by restoring the normal expression of genes involved in these pathways.

While synthetic circRNA sponges hold considerable promise, several challenges must be addressed for their effective therapeutic application. The efficiency of circRNA synthesis is notably impacted by RNA size. Larger circRNAs, especially those exceeding 500 nucleotides, pose significant production challenges with enzymatic ligation due to reduced circularization efficiency and increased steric hindrance. Exploring alternative methods, such as chemical ligation, may be necessary to overcome these limitations and facilitate the synthesis of larger circRNAs. The choice between natural and chemically modified nucleotides is crucial. While natural circRNAs are biologically relevant, chemical modifications can enhance stability, binding affinity, or specificity. However, modified circRNAs may also trigger unwanted immune responses or interfere with endogenous RNA processes, complicating their clinical application. The method of circRNA production—whether in vitro or in vivo—affects their functionality. In vitro methods offer precise control over circRNA composition and structure, whereas in vivo systems may better replicate natural cellular environments, potentially enhancing therapeutic relevance.

A balanced approach between in vitro and in vivo methods is essential for optimizing circRNA sponge efficiency. The efficient delivery of synthetic circRNA sponges to target tissues presents a significant challenge, as non-coding RNAs do not engage with conventional protein synthesis pathways. Advanced delivery systems, such as nanoparticles or viral vectors, are necessary to ensure precise cellular targeting. Strategies to minimize off-target effects and enhance bioavailability, particularly in specific tissues like the heart, are crucial for therapeutic success. The ability of circRNA sponges to regulate miRNAs offers a promising therapeutic avenue for targeting various diseases.

## 6. CircRNA Delivery Systems for Therapeutic Applications

The successful implementation of circRNA-based therapies relies heavily on the development of efficient delivery systems. Given their unique structure and therapeutic potential, circRNAs necessitate highly targeted delivery methods to ensure they reach the appropriate tissues and cells [78]. Effective delivery systems are essential for preserving the stability, specificity, and efficacy of circRNA therapeutics, as they protect the RNA from degradation and facilitate cellular uptake at the target site [79]. By optimizing these delivery mechanisms, we can enhance the therapeutic impact of circRNA-based interventions.

Delivering nucleic acids, including circRNAs, into target cells poses several challenges. CircRNAs, being relatively large molecules, have a circular structure that, while conferring stability, complicates conventional delivery methods. Additionally, nucleic acids are vulnerable to degradation by nucleases in the bloodstream, which limits their bioavailability [80]. Immune system activation can lead to unintended inflammatory responses. For circRNAs to exert their biological effects, they must efficiently traverse cellular membranes and avoid endosomal entrapment. Addressing these challenges is critical for optimizing circRNA therapeutics [81]. Effective delivery systems are essential, as they protect circRNAs from degradation, enhance target specificity, and improve therapeutic efficacy [82]. Ideal delivery systems must (1) safeguard circRNAs against enzymatic breakdown during circulation; (2) facilitate targeted delivery to specific tissues or cells, minimizing off-target effects; and (3) enable efficient intracellular delivery to ensure circRNAs reach their intended cellular compartments. In this context, both viral and non-viral delivery systems, along with advanced nanocarriers, represent promising strategies for the therapeutic application of circRNAs [83].

### 6.1. Mechanisms and Strategies for CircRNA Delivery

CircRNA delivery primarily utilizes two strategies: viral and non-viral delivery systems. Each has distinct advantages and limitations regarding efficacy, safety, and production ease. Viral delivery systems, such as adenoviruses and lentiviruses, are recognized for their high transduction efficiency and capacity to deliver large genetic constructs. However, they may pose safety concerns related to immune responses and potential insertional mutagenesis. In contrast, non-viral delivery systems—including liposomes, polymeric nanoparticles, and inorganic nanoparticles—provide a more versatile and generally safer alternative. These systems can be engineered to enhance stability, target specificity, and cellular uptake, although they may exhibit lower transfection efficiencies compared to viral methods. Ultimately, the choice of delivery strategy depends on the therapeutic context and the specific characteristics of the circRNA targeted. Optimizing these delivery mechanisms is essential for maximizing the therapeutic potential of circRNAs.

#### 6.1.1. Viral Delivery Systems

Adeno-associated viruses (AAVs) are widely employed in gene therapy due to their low immunogenicity and high transduction efficiency [84]. Their ability to stably express transgenes in both dividing and non-dividing cells makes AAVs particularly suitable for circRNA delivery [85]. AAVs have demonstrated success in targeting various tissues, including the heart, brain, and muscle, which is especially beneficial for applications in cardiovascular disease [86,87,88,89]. For instance, the AAV9-mediated cardiac overexpression of circFNDC3B in post-myocardial infarction (MI) hearts has been shown to reduce cardiomyocyte apoptosis, enhance neovascularization, and improve left ventricular function [42]. Additionally, AAVs have been utilized to deliver circmiRs to cardiomyocytes in vivo within a transverse aortic constriction (TAC) mouse model of cardiac disease [90]. Furthermore, the AAV9 vector-based overexpression of the well-conserved circITCH has been found to mitigate cardiotoxicity in mice [91].

Lentiviral vectors are notable for their ability to integrate genetic material into the host genome, facilitating the long-term expression of circRNAs [92]. This characteristic makes lentiviral vectors particularly advantageous for treating chronic conditions that require sustained circRNA expression [93]. However, the capacity for genomic integration also raises concerns regarding insertional mutagenesis, which could potentially lead to unintended effects, such as oncogenesis [94]. Consequently, while lentiviral vectors present a viable option for circRNA delivery, their long-term safety must be carefully evaluated in clinical applications [95]. Balancing the benefits of sustained circRNA expression with the associated risks will be essential in determining the suitability of lentiviral vectors for therapeutic use.

#### 6.1.2. Non-Viral Delivery Systems

Lipid nanoparticles (LNPs) have gained recognition for their effectiveness in nucleic acid delivery, particularly in encapsulating large RNA molecules and protecting them from degradation [96]. Their design enables targeted delivery to specific tissues and cells, which is crucial for maximizing the therapeutic efficacy of circRNAs. The successful implementation of LNPs in mRNA vaccines highlights their potential as an effective delivery system for circRNA-based therapeutics. By leveraging their ability to shield RNA from enzymatic degradation and enhance cellular uptake, LNPs offer a controlled and safe means of delivering circRNAs across various therapeutic contexts [96]. For instance, the delivery of recombinant circZFPM2 using LNPs has been shown to rescue cardiomyocyte hypertrophic gene expression and promote cell survival [97]. This demonstrates the capability of LNPs to facilitate meaningful biological outcomes, reinforcing their role as a promising tool in circRNA therapeutics.

Polymeric nanoparticles, composed of biodegradable polymers such as poly(lactic-co-glycolic acid) (PLGA), are engineered to release RNA in a controlled manner [98]. These nanoparticles are characterized by sustained release profiles, which can enhance the therapeutic efficacy of RNA over extended periods [99]. The versatility of polymeric nanoparticles enables them to encapsulate a variety of therapeutic agents, making them valuable tools in combination therapies. By facilitating targeted and gradual RNA release, polymeric nanoparticles can improve treatment outcomes in conditions requiring the precise modulation of gene expression [100]. Their capability for tailored delivery highlights their significance in advancing circRNA-based therapeutics for future applications.

Exosomes, naturally occurring extracellular vesicles, are increasingly recognized as effective vehicles for delivering circRNAs [101,102,103,104]. Their ability to cross biological barriers, such as the blood–brain barrier, allows exosomes to deliver cargo with high specificity. As cell-derived carriers, exosomes are biocompatible and less likely to provoke immune responses, making them an appealing option for the targeted delivery of circRNAs to specific tissues or organs [105]. Research has demonstrated several successful applications of exosomes in various disease contexts. For instance, the transfer of circDIDO1 mediated by mesenchymal stem cell (MSC)-isolated exosomes was shown to suppress hepatic stellate cell (HSC) activation, providing new insights into the prevention of liver fibrosis in patients with liver failure [106]. Additionally, an exosome-based delivery system effectively targeted mitochondria, delivering circRNA mSCAR preferentially to macrophages, which reduced systemic inflammation and alleviated mortality in sepsis [107]. Furthermore, circDIDO1-loaded exosomes have been reported to repress tumorigenicity and aggressiveness in gastric cancer [108].

#### 6.1.3. Physical Methods

Electroporation is a technique that uses an electrical field to temporarily increase the permeability of cell membranes, facilitating the entry of circRNAs into cells [109]. While this method is highly effective for in vitro applications, it poses challenges for in vivo delivery due to its invasive nature and potential to cause cell damage [110]. Microinjection, another physical delivery method, involves the direct introduction of RNAs into cells using fine needles [111]. This technique allows for precise control over the delivery process and is effective for research applications [112]. However, microinjection is labor-intensive and invasive, making it impractical for large-scale therapeutic use. Both electroporation and microinjection present unique advantages and limitations, emphasizing the need for alternative delivery strategies that combine efficiency with minimal invasiveness for the therapeutic applications of circRNAs.

### 6.2. Challenges in CircRNA Delivery

Despite advancements in circRNA delivery technologies, several significant challenges persist. (1) Tissue-Specific Targeting: Achieving the precise targeting of circRNAs to specific tissues while minimizing off-target effects remains a major hurdle in effective delivery. (2) Immune Response: Some delivery systems, particularly viral vectors, can elicit immune responses, which may limit their therapeutic applications and efficacy. (3) Long-Term Stability: Ensuring the long-term stability and sustained expression of therapeutic circRNAs is crucial for effectively treating chronic conditions. (4) Efficient Intracellular Delivery: Overcoming endosomal entrapment and ensuring that circRNAs reach their intended intracellular targets are critical challenges that must be addressed.

To tackle these issues, researchers are developing multifunctional nanocarriers capable of simultaneously delivering RNAs and other therapeutic agents. These carriers can be engineered to release various therapeutic cargos, including circRNAs, miRNAs, and small-molecule drugs, thereby enhancing the synergistic effects of combination therapies. Additionally, novel delivery methods, such as cell-penetrating peptides and hybrid delivery systems, are being explored to improve RNA uptake and bioavailability. By customizing circRNA formulations and delivery strategies based on individual genetic profiles and disease states, healthcare providers could offer precision therapies that improve efficacy while reducing side effects. The integration of circRNA therapies into personalized medicine has the potential to revolutionize the treatment of cardiovascular disorders.

## 7. Limitations and Future Directions

Despite the growing interest in circRNAs as potential therapeutic targets, there are significant limitations in the current body of research. Most findings regarding the roles of circRNAs, particularly in cardiovascular diseases, are derived from preclinical studies, often using animal models or in vitro systems. While these studies provide valuable insights into the molecular mechanisms of circRNAs, their direct relevance to human physiology remains uncertain. The therapeutic implications of targeting circRNAs, including examples like circSAMD4A, are largely hypothetical at this stage. Although there is evidence to suggest that circRNAs can influence processes such as apoptosis, inflammation, and cell proliferation, these findings have not yet been validated in clinical settings. No circRNA has been conclusively shown to possess diagnostic or therapeutic value in human cardiovascular diseases. Moreover, the transition from animal models to human applications poses significant challenges, including differences in circRNA expression profiles across species and the complexity of human cardiovascular diseases.

To move forward, further validation through comprehensive preclinical studies and well-designed clinical trials is essential. These studies should focus on establishing the safety, efficacy, and specificity of circRNA-based interventions. Additionally, the development of effective delivery systems for circRNA-targeting therapies remains a critical hurdle to overcome before any potential clinical applications can be realized. In summary, while circRNAs hold considerable promise as therapeutic targets, much work remains to be undertaken before their full potential can be harnessed. At present, their application in cardiovascular medicine is speculative, and more robust evidence is needed to support their clinical utility.

## 8. Conclusions

CircRNAs are emerging as crucial regulators of gene expression with significant therapeutic potential in CVDs. Their inherent stability and functional versatility position them as promising candidates for novel therapeutic interventions. Recent advancements in circRNA sponge synthesis have demonstrated their ability to modulate miRNA activity and gene expression with precise targeting. However, for circRNA-based therapies to succeed, effective and specific delivery systems are essential. Key challenges include achieving tissue-specific targeting, mitigating immune responses, and ensuring long-term stability, which are critical hurdles for successful clinical translation. Our group has explored the role of circRNAs in cardiovascular diseases, including pulmonary hypertension (PH), where circRNAs have shown potential as both biomarkers and therapeutic targets. Our research has revealed that circRNAs can regulate disease processes by influencing ion channels, cell proliferation, and vascular remodeling in PH. These findings highlight the broader applicability of circRNAs across various cardiovascular conditions [10,11,12].

Looking ahead, the most promising next steps involve optimizing delivery strategies, particularly through the development of tissue-specific systems that minimize off-target effects and enhance the therapeutic efficacy of circRNAs. Additionally, understanding the long-term impact of circRNA modulation on gene expression and potential immune reactions will be crucial for the safe and effective application of these therapies. Future researchers will likely face challenges in balancing therapeutic potency with immune safety and ensuring the durability of effects in vivo.

In summary, circRNA-based therapies hold considerable promise for personalized medicine, particularly in the cardiovascular field. By integrating circRNA technologies into clinical practice, we can develop targeted treatment strategies that address the underlying mechanisms of disease, thereby improving patient outcomes and minimizing side effects. Continued advancements in circRNA synthesis and delivery systems will undoubtedly pave the way for the next generation of circRNA-based therapies, offering new hope for complex cardiovascular conditions and transforming modern medicine.

## Figures and Tables

**Table 1:** CircRNAs involved in cardiovascular diseases.

CircRNA	Target miRNA	Function	Cardiovascular Disease	Ref.
circSAMD4A	miR-138-5p	Increases apoptosis and inflammation	Hypoxia/Reoxygenation (H/R) injury	[24]
circITCH	miR-17-5p	Inhibits Wnt/β-catenin pathway	Myocardial apoptosis	[25]
circNCX1	miR-133a-3p	Promotes oxidative-stress-induced apoptosis	Ischemic myocardial injury	[26]
MFACR	-	Stimulates mitochondrial division and apoptosis	Myocardial infarction	[27]
Circ-Amotl1	-	Activates Akt signaling pathway to enhance cell survival and regeneration	Doxorubicin (DOX)-induced cardiomyopathy	[28]
circPan3	miR-31-5p	Exacerbates cardiotoxicity via miR-31-5p/QKI axis	Doxorubicin (DOX)-induced cardiomyopathy	[29]
circSlc8a1	miR-133	Protects cardiac remodeling and reduces hypertrophy	Cardiac hypertrophy	[33,34,35]
circRNA_000203	miR-26b-5p	Stimulates myocardial fibrosis	Myocardial fibrosis	[49]
circHRCR	miR-223	Mitigates hypertrophy	Cardiac hypertrophy	[36]
circFNDC3b	mmu-miR-93-3p, mmu-miR-412-3p, mmu-miR-298-5p	Promotes cardiac repair	Myocardial infarction	[42]
circNfix	-	Promotes cardiomyocyte proliferation and angiogenesis and inhibits apoptosis	Myocardial infarction	[43]
circNFIB	miR-433	Reduces fibroblast proliferation and differentiation	Myocardial fibrosis	[19,50]
MICRA	-	Biomarker for predicting heart failure post myocardial infarction	Heart failure	[56,57]
